# Altered Spontaneous Brain Activity Patterns in Children With Strabismic Amblyopia After Low-Frequency Repetitive Transcranial Magnetic Stimulation: A Resting-State Functional Magnetic Resonance Imaging Study

**DOI:** 10.3389/fnhum.2022.790678

**Published:** 2022-04-08

**Authors:** Yi-Ning Wang, Yi-Cong Pan, Hui-Ye Shu, Li-Juan Zhang, Qiu-Yu Li, Qian-Min Ge, Rong-Bin Liang, Yi Shao

**Affiliations:** Department of Ophthalmology, The First Affiliated Hospital of Nanchang University, Jiangxi Center of National Ocular Disease Clinical Research Center, Nanchang, China

**Keywords:** low-frequency rTMS, strabismic amblyopia, amplitude of low-frequency fluctuation (ALFF), spontaneous brain activity, ophthalmological

## Abstract

**Objective:**

Previous studies have demonstrated altered brain activity in strabismic amblyopia (SA). In this study, low-frequency repetitive transcranial magnetic stimulation (rTMS) was applied in children with strabismic amblyopia after they had undergone strabismus surgery. The effect of rTMS was investigated by measuring the changes of brain features using the amplitude of low-frequency fluctuation (ALFF).

**Materials and Methods:**

In this study, 21 SA patients (12 males and 9 females) were recruited based on their age (7–13 years old), weight, and sex. They all had SA in their left eyes and they received rTMS treatment one month after strabismus surgery. Their vision before and after surgery were categorized as pre-rTMS (PRT) and post-rTMS (POT). All participants received rTMS treatment, underwent magnetic resonance imaging (MRI), and their data were analyzed using the repeated measures *t*-test. The team used correlation analysis to explore the relationship between logMAR visual acuity and ALFF.

**Results:**

Pre- versus post-rTMS values of ALFF were significantly different within individuals. In the POT group, ALFF values were significantly decreased in the Angular_R (AR), Parietal_Inf_L (PIL), and Cingulum_Mid_R (CMR) while ALFF values were significantly increased in the Fusiform_R (FR) and Frontal_Inf_Orb_L(FIL) compared to the PRT stage.

**Conclusion:**

Our data showed that ALFF recorded from some brain regions was changed significantly after rTMS in strabismic amblyopes. The results may infer the pathological basis of SA and demonstrate that visual function may be improved using rTMS in strabismic amblyopic patients.

## Introduction

**FIGURE 1:**
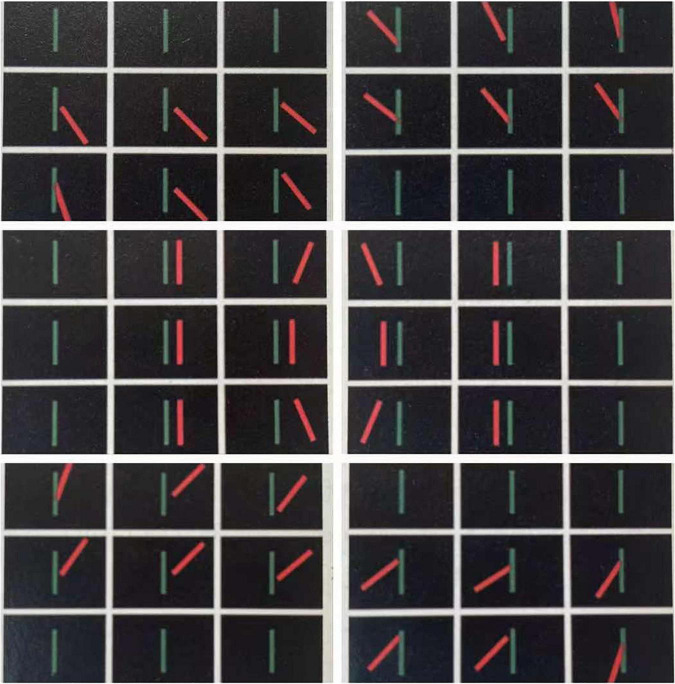
Diplopia in each of the external ocular muscles with paralysis.

Strabismus and amblyopia are two common visual developmental disorders that can occur in infancy and persist into adulthood if treatment is not successful (Tarczy-Hornoch et al., 2013; Chen et al., 2014). Strabismus is an optical manifestation disorder associated with the coordination of the external eye muscles. Different conditions of extraocular muscle incongruity may result in different types of diplopic images (Figure 1). Strabismus is generally considered to be associated with maldevelopment of the visual pathways in the brain that mediate eye movement (Min et al., 2018), and inimically affects stereopsis, binocularity, and depth perception (Gunton et al., 2015). Hyperopia, muscle dysfunction, trauma, brain disease, and infection are all important causes of strabismus, and risk factors include premature birth and cerebral palsy. Diagnosis can be made by the observation of light reflected from the anterior eye offset from the center of the pupil. Treatments, including refractive correction and eye alignment surgery, address the impact on vision and are good choices for patients. The incidence rate of adult-onset strabismus is reportedly 54.2 per 100,000 individuals (Mohney et al., 2012), and in adults strabismus is often associated with undiagnosed amblyopia in early childhood and symptoms associated with aging (Chan et al., 2004).

Amblyopia, also known as lazy eye, is a visual disorder with causes which affect cooperation between the eyes and brain (National Eye Institute, 2016). Because the condition is associated with changes in the brain, visual impairment such as ametropia cannot be fully treated after surgery (Schwartz et al., 2002; Levi, 2013). Early detection can improve the rate of successful treatment (Jefferis et al., 2015) and glasses may be an important part of treatment for children (Jefferis et al., 2015; Maconachie and Gottlob, 2015). Many patients with amblyopia, especially mild cases, are unaware of their condition because one eye is affected and the vision of the fellow eye is normal. However, because two eyes are needed for stereo vision (which is usually lacking for the patients), patients with amblyopia may have relatively poor vision, and relatively low sensitivity to contrast and movement in the affected eye (Fazzi and Bianchi, 2016). Amblyopia features reduced stereopsis, visual acuity (VA), visual sensitivity, and impaired spatial vision and binocular summation (Webber and Wood, 2005).

Diplopia refers to the abnormal phenomenon of one object being perceived as two identical but separated images. There are many causes of diplopia, some of which reflect anomalies of the brain (Cerulli Irelli et al., 2021). Diplopia may occur when one or more of the six muscles that control eye movement become inflamed, injured, or neurologically impaired, and the muscles in both eyes do not accurately coordinate (Goseki et al., 2021). Because strabismus causing diplopia and confusion (perceived superposition of two different images) can make the patient feel unwell, the image from the macula of the strabismic eye may be suppressed for a sustained period, and the amblyopia that accompanies this situation is known as strabismic amblyopia (Peña Urbina et al., 2021).

Magnetic resonance imaging (MRI) has been widely used in various pretreatment imaging diagnoses recently. As a non-invasive neuroimaging method, it is mainly used to evaluate the functional and structural changes of the human brain (Brown et al., 2016). Functional Magnetic Resonance Imaging (fMRI) is a technology with precise spatial resolution and is used for the analysis of brain function (Goodyear and Menon, 2001). This imaging method helps to reveal the mechanism underpinning eye diseases (Conner et al., 2007; Wang et al., 2012; Liang et al., 2017). Repetitive transcranial magnetic stimulation (rTMS) mainly achieve excitation or inhibition of local cerebral cortical function by changing the stimulation frequency, with the aim of effective treatment of disease. Thompson’s present data show that rTMS of the visual cortex can temporarily improve contrast sensitivity in the adult amblyopic visual cortex (Thompson et al., 2008). Spiegel have shown that a-tDCS to the visual cortex would improve contrast sensitivity in adult patients with amblyopia by enhancing the cortical response to inputs from the amblyopic eye (Spiegel et al., 2013). Rehn found that rTMS could improve the symptoms of patients with obsessive-compulsive disorder (Rehn et al., 2018). Chou demonstrated the effect of rTMS on Alzheimer’s disease (Chou et al., 2020). TMS can be used to stimulate the cerebral cortex, such as the visual cortex and somatosensory cortex, to induce local excitatory or inhibitory effects and affect the function of the system. In addition, TMS can also be used in learning, memory, language, and emotional research (Brunoni et al., 2017; Sebastianelli et al., 2017; McClintock et al., 2018; Arns et al., 2019; Yang et al., 2019; Chou et al., 2020; Malkani and Zee, 2020).

Studies suggested the neural networks modulating aspects of emotional behavior to be implicated in the pathophysiology of mood disorders. These networks involve the prefrontal cortex (Rigucci et al., 2010). AhmedA Abdelrahman’s findings revealed that HF-rTMS over L-DLPCF for 10 days reduced cigarette consumption, craving, dependence, and improved associated symptoms of anxiety and depression (Abdelrahman et al., 2021). The abnormal activity of dorsolateral prefrontal cortex (DLPFC) is related to the occurrence of anxiety and depression.

## Materials and Methods

### Subjects

Twenty-one patients (12 males and 9 females) were recruited at the First Affiliated Hospital of Nanchang University. Those eligible for participation met the following criteria: (1) Aged between 7 and 13 years old; (2) Their guardian allowed them to receive rTMS treatment within 1 month after strabismus surgery; (3) Clear diagnosis of strabismus. Exclusion criteria were as follows: (1) Patients suffering from other eye diseases, such as cataract, glaucoma, or retinitis pigmentosa; (2) Mental illness. The 21 patients who met the criteria underwent rTMS treatment before and after surgical treatment. The Medical Ethics Committee of the First Affiliated Hospital of Nanchang University approved the research plan. Patients and guardians were informed about the research and potential risks before signing consent forms voluntarily.

This study sought consent from all patients participating in the diagnosis, treatment, and evaluation stages of the study.

### Transcranial Magnetic Stimulation

Repetitive transcranial magnetic stimulation (rTMS) was administered by trained researchers using the Magstim Rapid device (Magstim^®^, Whitland, Wales, United Kingdom) and Magstim d70-mm-air-cooled figure-of-eight coil. Use of stimulus frequency rate of 10Hz, intensity of 100% resting motion threshold, 5s stimulation, pulse 50/5s, the interval was 10s, with 2000 pulses in total, and the treatment lasted for 10min. The stimulation site is DLPFC. Stimulation was performed between baseline measurements and remeasurements. The two rTMS stimulation groups received 30 treatments 5 times a week for 6 weeks.

### Magnetic Resonance Imaging Parameters

All patients underwent scanning using 3-TESLA MR scanners (Siemens, Germany) and a gradient-echo echo-planar imaging pulse sequence was used to acquire fMRI values with the following specific parameters: 240 functional images (repetition time = 2,000 ms, echo time = 30 ms, thickness = 4.0 mm, gap = 1.2 mm, acquisition matrix = 64 × 64, flip angle = 90°, field of view = 220 × 220 mm, 29 axial) were obtained. All MRI images were examined for structural abnormalities, and no subject was excluded on this basis.

### Functional Magnetic Resonance Imaging Data Processing

The CAT12 toolkit (12.7^1^) from the Statistical Parametric Mapping database (SPM12^2^) was used to analyze the data. All procedures were performed using MATLAB 7.9.0 software (R2009b; The Mathworks, Inc., Natick, MA, United States). Preprocessing included calibration, correction for head movement, image structure and average echo planar imaging alignment, normalization to a standard template, and smoothing using a Gaussian of 8 mm full width at half maximum. The fMRI brain functional images of each subject were co-registered with the T1 brain structure image template of the Montreal Neurological Institute (MNI) space as the reference standard, and the spatial standardization was completed using the Diffeomorphic Anatomical Registration Through Exponentiated Lie algebra (DARTEL) method. In addition, the gray matter volumes were normalized and smoothed using a 6-mm full width at half maximum Gaussian kernel.

### Correlation Analysis

LogMAR acuity tests were used to assess the patients’ visual monocular acuity. The correlations between rTMS and logarithmic MAR values in the AR (*P* = 0.0066), FR (*P* < 0.0001), and CMR (*P* = 0.0004) regions were analyzed using GRAPHIPAD Prism 8. Correlation graphs were produced.

### Statistical Analysis

After controlling for age and sex, the repeated measures *t*-test was used to compare the amplitude of low-frequency fluctuation (ALFF) between PRT and POT. The significance level was set at *P* < 0.05, family wise error corrected, voxel level *P* < 0.001, and cluster level *P* < 0.05. A color map was created by overlapping the significant voxels by standardization of 3-dimensional magnetization prepared fast acquisition gradient echo sequences.

## Results

### Demographics and Visual Measurements

There were no significant differences in age and log MAR-R(BCVA) between the POT and PRT. There were statistically notable differences in the log MAR-L(BCVA) (*P* < 0.05) between the two groups (more details are presented in Table 1).

**TABLE 1:** Demographics and clinical measurements of pre-rTMS (PRT) and post-rTMS (POT).

Condition	PRT	POT	t/x2	P
Male/female	12/9	12/9	N/A	N/A
Age (year)	9.16 ± 2.42	9.16 ± 2.42	N/A	N/A
Weight (Kg)	22.36 ± 7.27	22.36 ± 7.27	N/A	N/A
SE-L (diopter)	3.75 ± 1.35	3.45 ± 1.55	4.431	0.864
SE-R (diopter)	3.15 ± 1.55	3.25 ± 1.35	4.064	0.809
Astigmatism-L (diopter)	1.50 ± 0.50	1.45 ± 0.55	5.873	0.912
Astigmatism-R (diopter)	1.55 ± 0.55	1.35 ± 0.45	5.054	0.846
Esotropia/exotropia	12/9	0/0	NA	NA
Color Vision	Full	Full	NA	NA
Confrontation visual field	Full	Full	NA	NA
log MAR-L(BCVA)	0.68 ± 0.15	0.43 ± 0.25	1.873	0.024
log MAR-R(BCVA)	–0.15 ± 0.05	–0.10 ± 0.05	7.439	0.927

*The 2-sample T-test was analyzed between the same patients before and after low frequency (LF) repetitive transcranial magnetic stimulation (rTMS); Data are expressed as mean ± standard deviation.*

### Differences in Amplitude of Low-Frequency Fluctuation

**FIGURE 2:**
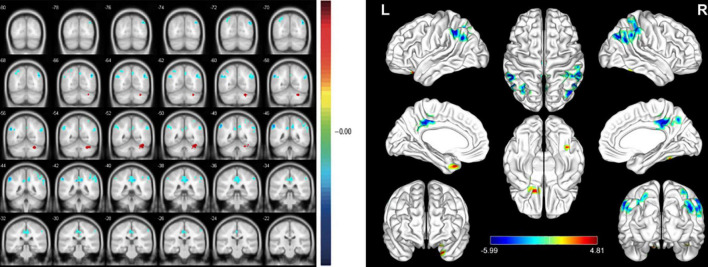
Significant differences in spontaneous brain activity between the pre-rTMS (PRT) and post-rTMS (POT). The sizes of the spots denote the degree of quantitative changes. The different brain regions were observed in the Angular_R (AR), Parietal_Inf_L (PIL), Cingulum_Mid_R (CMR), Fusiform_R (FR) and Frontal_Inf_Orb_L (FIL). The red areas denote that PRT exhibit higher amplitude of low-frequency fluctuation (ALFF) in brain areas than POT and the blue areas denote brain regions with a lower ALFF [*P* < 0.001, cluster > 13 voxels, Alphasim corrected)].

At the PRT stage, the ALFF values were significantly higher in the AR, PIL, and CMR [Figure 2 (red) and Table 2]. and were significantly lower in the FR and FIL compared to the POT stage [Figure 2 (blue), 3 and Table 2]. No significant difference was found in ALFF between PRT and POT in other brain regions (P > 0.05).

**TABLE 2:** Brain areas with significantly different amplitude of low-frequency fluctuation (ALFF) between groups.

Brain areas	MNI coordinates			number of voxels	T value
	X	Y	Z		
PRT < POT					
Fusiform Gyrus (R)	39	–51	–24	61	4.192
Frontal Inf Orb Lobe (L)	–21	21	–27	43	4.8077
PRT > POT					
Angular Gyrus (R)	45	–66	36	240	–5.6255
Cingulum Mid Lobe (R)	9	–39	39	224	–5.0931
Parietal Inf Lobe (L)	–54	–45	45	127	–5.9884

*The statistical threshold was set at the voxel level with P < 0.05 for multiple comparisons using False Discovery Rate (Q < 0.01 and cluster size > 15). ALFF, amplitude of low-frequency fluctuation; L, left; R, right; MNI, Montreal Neurological Institutet; PRT, pre-rTMS; POT, post-rTMS.*

**FIGURE 3:**
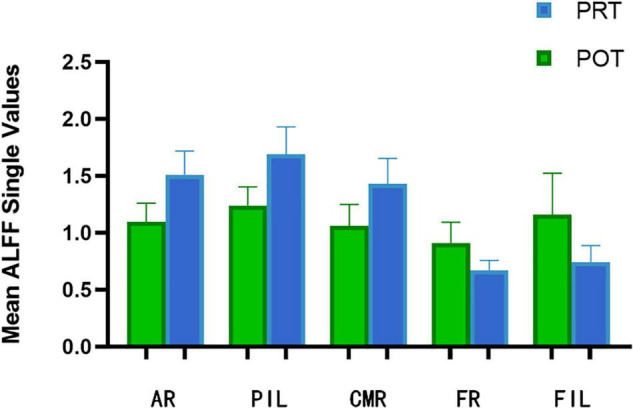
The mean ALFF values between POT and PRT. ALFF, amplitude of low-frequency fluctuation; PRT, pre-rTMS; POT, post-rTMS; L-left; R-right; AR, Angular_R; PIL, Parietal_Inf_L; CMR, Cingulum_Mid_R; FR, Fusiform_R; FIL, Frontal_Inf_Orb_L.

### Correlation Analysis

**FIGURE 4:**
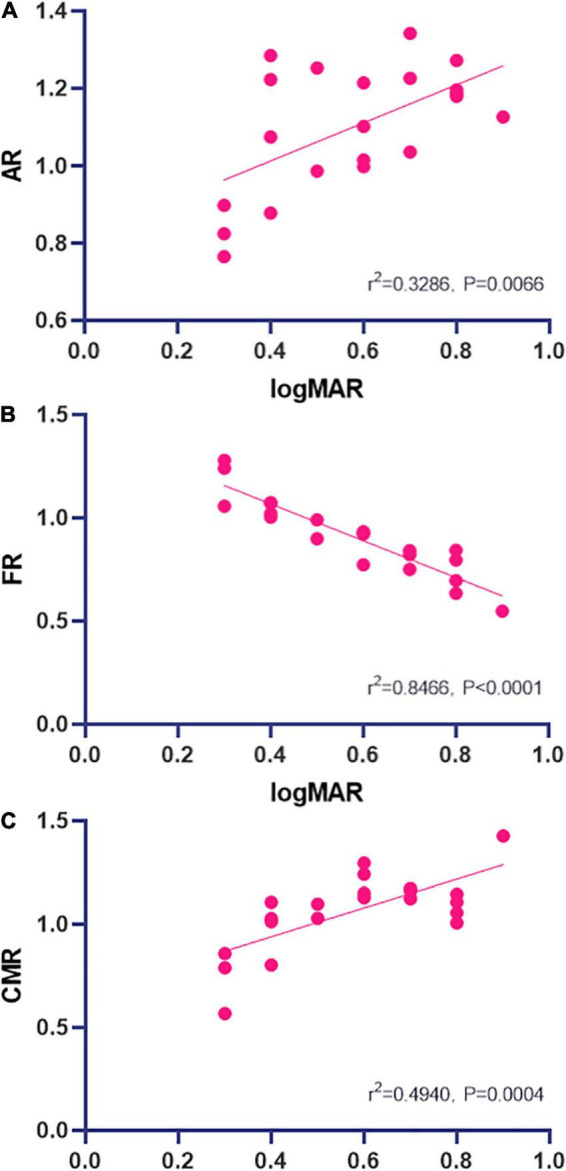
Correlations between mean ALFF signal values and logMAR acuity. The ALFF signal value of AR showed a positive correlation with logMAR [*r*^2^ = 0.3286, *P* = 0.0066; **(A)**] and the ALFF signal value of FR showed a negative correlation with logMAR [*r*^2^ = 0.8466, *P* < 0.0001; **(B)**]. The logMAR showed a positive correlation with the ALFF signal value of CMR [*r*^2^ = 0.4940, *P* = 0.0004; **(C)**] (A lower LogMAR value means better acuity). ALFF, amplitude of low-frequency fluctuation; AR, Angular_R; CMR, Cingulum_Mid_R; FR, Fusiform_R.

**FIGURE 5:**
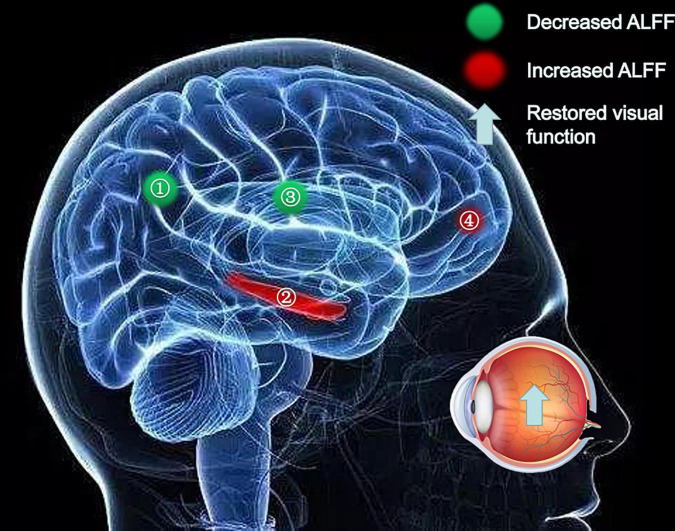
Significant differences in spontaneous brain activity between the PRL and POL stages. Different brain regions that were observed: (1) Angular_R and Parietal_Inf_L; (2) Fusiform_R; (3) Cingulum_Mid_R; (4) Frontal_Inf_Orb_L. The red areas indicate increased ALFF values, and the green areas indicate decreased ALFF values. ALFF, amplitude of low-frequency fluctuation; L, left; R, right.

**FIGURE 6:**
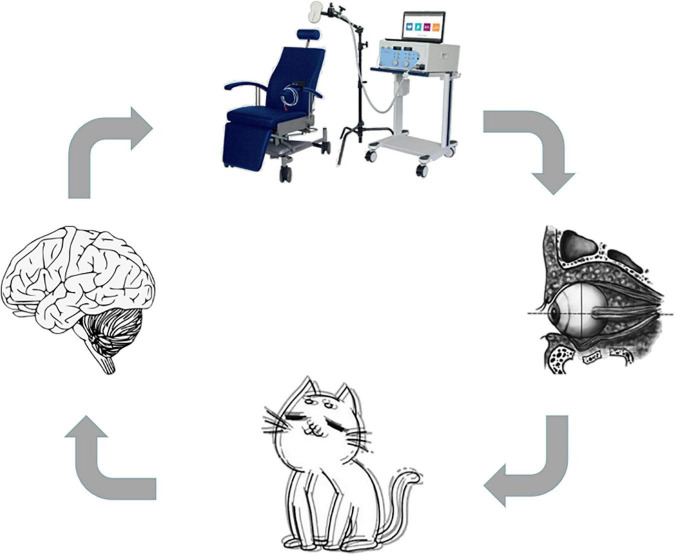
Relationship between rTMS and diplopia. Patients with SA may develop diplopia after surgery. Once diplopia occurs, visual function may be affected, leading to abnormal neural activity in brain regions. SA, oblique amblyopia disease.

At the POT stage, significant correlations between ALFF signal value and logMAR acuity were found and were positive at AR (*r*^2^ = 0.3286, *P* = 0.0066; Figure 4A) and negative at FR (*r*^2^ = 0.8466, *P* < 0.0001; Figure 4B) and positive at CMR (*r*^2^ = 0.4940, *P* = 0.0004; Figure 4C). Changes in ALFF values in brain regions are associated with changes in visual acuity and brain activity (Figures 5, 6).

### Receiver Operating Characteristic Curves

**FIGURE 7:**
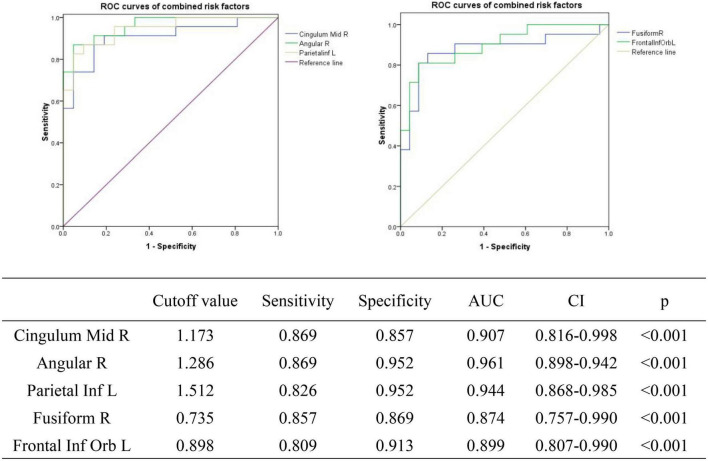
ROC curve analysis of the mean ALFF values of the affected brain regions in SA patients. ROC, receiver operating characteristic; AUC, area under the curve; CI, confidence interval; ROC, receiver operating characteristic; ALFF, amplitude of low-frequency fluctuation.

We tested the hypothesis that ALFF may be a potential diagnostic indicator of SA patients at the PRT stage by plotting the average ALFF values of each brain region on an ROC curve. Brain regions with significantly different ALFF values showed high accuracy as diagnostic markers (PRT > POT). The AUCs of the ALFF values of the different brain regions were as follows: CMR (0.907, *p* < 0.001), AR (0.961 *p* < 0.001), PIL (0.944, *p* < 0.001), FR (0.874, *p* < 0.001), FIL (0.899, *p* < 0.001) (Figure 7).

## Discussion

Amplitude of low-frequency fluctuation (ALFF) is a commonly used clinical method which reflects changes in spontaneous brain activity and has been applied in the investigation of several ophthalmological diseases. Amplitude of low-frequency fluctuation is used to measure spontaneous fluctuations in blood oxygen level-dependent fMRI-signal intensity for nervous activity, reflecting the intensity of regional spontaneous brain activity at rest. The increase of ALFF indicated increased blood oxygen dependence level and increased activity in this brain region, on the contrary, activity of the brain area decreased. We used the ALFF sequence to compare the activity of different brain regions in POT and PRT. Our study is the first to determine whether there are ALFF differences in brain regions before and after transcranial magnetic stimulation within SA patients after surgery, and to identify those regions. Functional MRI is one of the most widely used functional brain imaging techniques. ALFF has been applied in ophthalmologic and neurogenic diseases, and in this study (Table 3) we demonstrated that the intrinsic patterns of activity in different brain regions of POT were changed after rTMS.

**TABLE 3:** Amplitude of low-frequency fluctuation (ALFF) method applied in Ophthalmologic and Neurogenic diseases.

	Author	Year	Disease
Ophthalmologic diseases	Hilbert et al., 2019	2016	Optic neuritis
	Wang and Luo, 2004	2018	Comitant strabismus
	Makris et al., 2013	2019	Acute eye pain
	Bocca et al., 2015	2019	SAvanced monocular blindness
	Li et al., 2020	2020	Retinal detachment
Neurogenic diseases	Ferreira et al., 2011	2011	Alzheimer’s disease
	Wang et al., 2020	2020	Parkinson’s disease
	Suzuki et al., 2020	2020	Huntington’s disease

We collected information on the brain regions where ALFF changed after rTMS treatment, including areas unrelated to visual processing (Table 4). There were significant differences in ALFF values in several brain areas between POT and PRT stages. Values were decreased in the FR and FIL regions, but increased in the AR, PIL, and CMR regions. These results demonstrate bidirectional changes in ALFF values between the POT and PRT stages. The ROC curve shows the clinical diagnostic significance of both the left parietal inferior, right cingulum middle, right angular, right fusiform, and left frontal inferior orb regions. The AUCs of all these brain areas are high, indicating that ALFF may be used in clinical diagnosis of other brain diseases in SA patients after surgery.

**TABLE 4:** Brain areas with altered amplitude of low-frequency fluctuation (ALFF) and potential impacts.

Brain areas	Experimental result	Brain function	Anticipated results
Cingulum Mid R	POT < PRT	Complex somatic and visceral motor function and pain response	Improve monitoring sensation and stereotactic and memory function
Angular R	POT < PRT	The production, expression and reception of language	Improve the ability to identify, explain, or remember words
Parietal inf L	POT < PRT	Ability in mathematics and logic	Improve logical thinking, divergent thinking and other aspects of the ability
Fusiform R	POT > PRT	Responsible for identifying the subcategories of objects	Improved ability to identify similar objects
Frontal Inf Orb L	POT > PRT	Responsible for thinking, calculation, and related to individual needs and emotions	Removal of schizophrenia, major depression and anxiety disorders

*PRT, pre-rTMS; POT, post-rTMS; L, left; R, right.*

Of these areas, the fusiform gyrus is located at the middle inferior part of the visual association cortex. The fusiform gyrus is not only used for face recognition, it is also responsible for the recognition of subcategories of objects (SparkNotes, 2007). In the fusiform face area, most studies have shown that the right hemisphere is more important for face recognition than the left. The face recognition area is located in the right fusiform gyrus, but it is involved in the recognition of a variety of complex stimuli and is a key brain region for acquiring the skills to distinguish similar objects (Tordesillas-Gutierrez et al., 2018). In patients with strabismic amblyopia, rTMS treatment significantly improved the condition of postoperative visual recovery, which may be related to the changes in the activity of the fusiform gyrus.

The angular gyrus, above Wernicke’s area, at the parietal-occipital junction, is an important association area in the back of the brain. If the angular gyrus is removed, the visual and auditory perception of words will lose connection, causing dyslexia (a reading and writing disorder) and audio-visual aphasia. In the latter, the sufferer loses the connection between what is seen and what is heard and is unable to understand the meaning of the words (Tordesillas-Gutierrez et al., 2018; Palejwala et al., 2020).

The middle part of the right cingulate is the gyrus between the cingulate sulcus of the medial part of the brain hemisphere and the sulcus of the corpus callosum, belonging to the cortical part of the limbic system (Creel, 2012). This brain region transmits nerve impulses to the anterior cingulate gyrus and striatum and receives output from the amygdala, orbitofrontal gyrus, and medial frontal gyrus (Li et al., 2017). It has long been understood as an important part of the emotional circuit, involved in processes such as emotion and self-evaluation, and is closely associated with depressive symptoms. Audiovisual hallucinations, sensory illusions, and emotional symptoms caused by diplopia onset usually occur after surgery and accompanied by a slow recovery period of 12 months. After rTMS treatment, the condition will be greatly reduced (Sasaki et al., 2016; Craciun et al., 2018). Primary epileptic seizures in the cingulate gyrus are mainly complex partial seizures with characteristic automaticity, autonomic nervous dysfunction, affective changes, and urinary incontinence. We speculate that rTMS can effectively control epilepsy in part of the cingulate gyrus brain region. SA may cause abnormalities in cerebral blood flow and metabolism in the brain, resulting in hypofunction, which in turn leads to depression in patients (Craciun et al., 2018).

The lower and upper parietal gyrus contain visual segmentation groups, which can be distinguished according to structural or connection standards, topographic organization, or other functional standards. The conventional view that this brain area is a type of visual sensory area and contains numerous optic motor neurons and parietal visual function focuses on its role in spatial perception, so the lesions in this area will cause abnormalities in the relevant visual field. Therefore, our research showing a decreased ALFF of the left parietal inferior region may manifest as a defect in the visual field, and this pathological change will seriously affect the daily life of the patient (Hilbert et al., 2019).

Previous studies have demonstrated a link between the frontal cortex and emotion. The occurrence and development of the clinical symptoms of schizophrenia, major depression, and anxiety depend on the frontal cortex system. Evidence suggests that affective function, experience, expression, and load information processing have different neural representations in the frontal cortex (Wang and Luo, 2004; Makris et al., 2013; Bocca et al., 2015). Therefore, it is reasonable to infer that the effect of rTMS on the activity of the frontal cortex may be useful for the treatment of patients with schizophrenia, major depression, and anxiety disorders.

We believe that ALFF has clinical significance, it can detect abnormal changes in the brain activity of patients through fMRI in advance of this factor, and provide timely interventions to effectively reduce the sequelae and complications of SA such as diplopia. It is worth noting that the limitations of the current study, including differences in measurement standards and other factors, require further unification of measurement scales in in-depth studies to verify the findings. The clinical features we used in this study were not rigorous. For example, different SA patients have different degrees of visual acuity recovery after surgery, some of which were not significantly different from the preoperative stage. Therefore, attention should be paid to these problems in future studies and the sample size should be expanded to accurately evaluate the changes of brain ALFF indexes in SA patients after surgery. In addition, the control group of patients treated with rTMS after SA surgery was not included in this study, changes in ALFF values generated by longitudinal brain recombination after SA surgery were not measured. As a result, in the results obtained in this study, the difference in ALFF value is not solely affected by rTMS treatment, and part of the difference may be related to longitudinal brain reorganization, the specific differences need to be further studied. Despite these deficiencies, this study revealed specific changes and effects of rTMS on ALFF in the brain regions of SA patients.

## Conclusion

In summary, we confirmed that within SA patients ALFF changes significantly in some brain regions after rTMS. Changes in ALFF reflect increases or decreases in activity within brain regions and may partly reflect the degree of improvement in visual dysfunction caused by postoperative complications in patients with SA. ALFF may be used for clinical diagnosis and evaluation of postoperative rehabilitation in patients with SA.

## Data Availability Statement

The raw data supporting the conclusions of this article will be made available by the authors, without undue reservation.

## Ethics Statement

All research methods were approved by the Committee of the Medical Ethics of the First Affiliated Hospital of Nanchang University and were in accordance with the 1964 Helsinki declaration and its later amendments or comparable ethical standards. All subjects were explained the purpose, method, potential risks and signed an informed consent form. Written informed consent to participate in this study was provided by the participants’ legal guardian/next of kin. Written informed consent was obtained from the individual(s), and minor(s)’ legal guardian/next of kin, for the publication of any potentially identifiable images or data included in this article.

## Author Contributions

Y-NW and Y-CP analyzed the data and drafted the manuscript. H-YS, Q-MG, and Y-CP assisted with data interpretation and figure composing. L-JZ, R-BL, and Q-YL collected the data. YS conceived, designed, and directed the study and finally revised and approved the manuscript.

## Conflict of Interest

The authors declare that the research was conducted in the absence of any commercial or financial relationships that could be construed as a potential conflict of interest.

## Publisher’s Note

All claims expressed in this article are solely those of the authors and do not necessarily represent those of their affiliated organizations, or those of the publisher, the editors and the reviewers. Any product that may be evaluated in this article, or claim that may be made by its manufacturer, is not guaranteed or endorsed by the publisher.

## Footnotes

1
http://www.neuro.uni-jena.de/cat/

2
https://www.fil.ion.ucl.ac.uk/spm/
